# Laser Doppler Blood-Flow Signals from Human Teeth during an Alignment and Leveling Movement Using a Superelastic Archwire

**DOI:** 10.1155/2013/102816

**Published:** 2013-09-19

**Authors:** Alvaro Wagner Rodrigues Salles, Adriana Mirian Cotrim Salles, Gessé Eduardo Calvo Nogueira

**Affiliations:** ^1^Instituto de Pesquisas Energéticas e Nucleares, IPEN-CNEN/SP, Avenida Professor Lineu Prestes 2242, 05508-000 São Paulo, SP, Brazil; ^2^University of Manitoba, Faculty of Dentistry, Preventive Dental Science, Pediatric Division, D341 Dental Bldg, 790 Bannatyne Avenue, Winnipeg, MB, Canada R3E 0W2

## Abstract

*Objective*. The purpose of this study was to examine alterations in blood-flow signals (BFS) from human teeth during an alignment and leveling phase (superelastic wire 0.014′′) in a clinical orthodontic treatment using laser doppler flowmetry (LDF). *Materials and Methods*. Recordings were made in 12 maxillary left central incisors. The basal value of the BFS from each tooth (without orthodontic forces) was compared with the corresponding values of BFS during four periods of observation: 20 minutes, 48 hours, 72 hours, and one month after the activation of the orthodontic appliance. *Results*. Statistically significant decrease of BFS was observed at 20 minutes, 48 hours, and 72 hours (*P* < 0.05). No differences were found comparing BFS on day 30 and the corresponding basal values. *Conclusion*. Under real clinical conditions, a significant decrease in BFS was verified during the initial phase of the treatment, followed by a recovery on day 30.

## 1. Introduction

A common reaction to orthodontic forces is an inflammatory process in the periodontal support tissues. The inflammatory process is a necessary condition for tooth movement and commonly affects the dental pulp [1]. Changes in blood flow are closely related to inflammatory processes. Thus, the evaluation of changes in the blood flow in the dental pulp and periodontal tissues during the application of orthodontic forces is of interest to study the mechanisms of the movement and related iatrogenic alterations in the dental pulp and root [1–4]. In initial studies, alterations in blood flow were analyzed in animal models by invasive and destructive methods due to the difficulties in accessing the pulp and periodontal tissues. More recently, the laser Doppler flowmetry (LDF) has been used to study practically all human organs. The method is noninvasive, offers no risk and allows measurement in real time. 

The blood-flow signals (BFS) from intact teeth, measured by the LDF, contain information of alterations in the blood flow in the dental pulp. Therefore, the technique has been used to evaluate alterations in blood flow in the dental pulp during orthodontic movements in humans. The information in the literature is limited to experimental movements such as intrusive (intermittent forces) [5–8], extrusive (intermittent forces) [5], intrusive (continuous forces during six days) [8], and tipping (continuous forces during three days) ones [9]. In these studies, experiments were carried out in laboratory, and the movements were monitored during short time intervals. BFS changes in real clinical time and conditions remain unknown. The lack of information is likely due to practical difficulties such as mechanical instabilities of the probe and the low spatial resolution of the LDF, resulting in contamination of the measurement from adjacent regions. The purpose of this study was to examine the effect of a superelastic archwire on BFS during an alignment and leveling phase of an orthodontic treatment under real clinical conditions, using a hybrid technique for long-term measurements.

## 2. Materials and Methods

### 2.1. Subjects

The institutional ethics committee on human research approved this study, and all participants signed an informed consent form. Twenty-three volunteers with class II division 1 malocclusion were preselected from patients scheduled for treatment at the Sociedade Paulista de Ortodontia e Ortopedia Facial. From this group, twelve patients, seven males, and five females, age 17.5 ± 3 years (mean ± SD), were included in the study. The inclusion criteria were accepting only patients with a healthy maxillary left central incisors and healthy periodontal tissues. Teeth were considered healthy only if they were free from caries and restorations and if there were no changes in their color. Periapical radiographs were taken of all patients to ensure that the morphology of the pulps and periodontal bone supports were within normal parameters. Patients with health problems or history of trauma were not considered for this study.

### 2.2. Blood Flow Measurement

A Laser Doppler Flowmeter (moorLab, Moor Instruments, UK) with a wavelength of 780 nm and a dental probe MP13 (Moor instruments, UK; 2 fibers, 0.25 mm diameter; centers 0.5 mm spaced apart) were used. The cutoff frequency of the LDF was set at 3.1 kHz. The flowmeter was calibrated according to the manufacturer's instructions. The data, obtained from the LDF, were recorded in computer as arbitrary perfusion units (AU) [10]. Room temperature was maintained from 20°C to 25°C. A rest of 30 minutes was provided to the volunteers prior to each session. 

### 2.3. Probe Stabilization and Tooth Isolation

**Figure 1:**
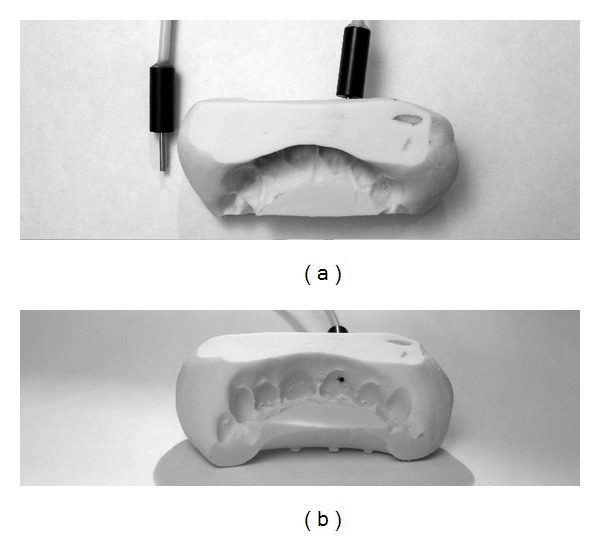
Silicone splint and probe. Silicone splint was used to maintain the probe mechanically stable on the tooth. (a) Top view and (b) side view.

**Figure 2:**
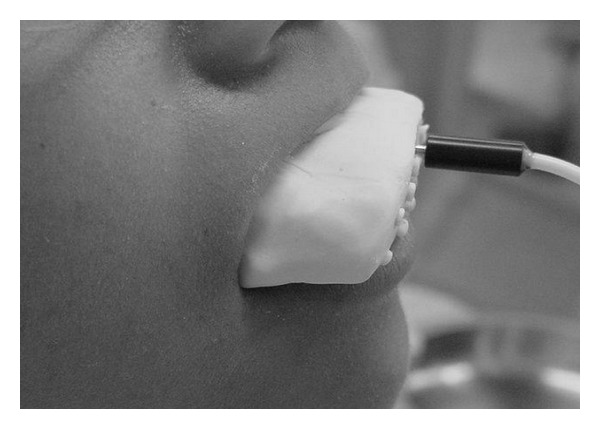
Laser Doppler probe stabilized by a silicone splint, applied in one volunteer.

**Figure 3:**
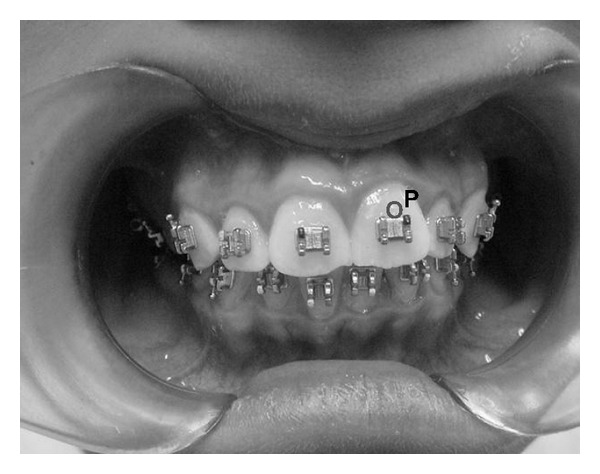
Brackets bonded to the teeth. The point (P) indicates the site where the probe was positioned, close to the bracket.

A silicone splint was used to stabilize the dental probe on the tooth, following the methodology proposed by McDonald and Pitt Ford [9], modified as follows. The retentive area of the bracket was covered with a layer of wax (Kota industry LTDA, Brazil). Afterwards, an impression of silicone was taken (Optosil comfort/Xantopren, Heraeaus Kulzer GmbH, Germany). Then, the impression of silicone was removed allowing visualization of the mark of the bracket. A hole was made above the mark of the bracket in the mold with a stainless steel drill (1.5 mm of diameter). This was done to allow the probe to pass through the mold and touch the teeth to be tested, as shown in Figures 1 and 2. The probe tip was positioned using the bracket as a reference, as shown in Figure 3. Therefore, it was assured that the probe was placed on the tooth at the same position every time the measurement was performed. The silicone splint was also used to (optically) isolate the measured tooth, to reduce contamination of the BFS from adjacent tissues [11].

### 2.4. Records during the Alignment and Leveling Phase

Orthodontic treatment plans for the selected volunteers were prepared according to each case separately. All the anterior teeth of the volunteers were slightly protruded. In all treatment plans, the first two stages were alignment and leveling of the teeth. In each patient, after the extraction of the first premolars, the protruded teeth were subjected to conventional alignment and leveling movements by means of a round 0.014-inch Ni-Ti wires (Round Sentalloy Accuform, Dentsply GAC International, NY, USA) for the first step (and other wires, not studied, for the second and further steps). Brackets with 0.022 inch slots were used. The wires above described were placed into the slots of the brackets and tied with stainless steel ligatures caliber 0.020 inch. Therefore, in this first step of the treatment, both leveling and alignment could not be differentiated, and the predominant orthodontic movements were tipping (labiolingual and mesiodistal), intrusion, or extrusion. The magnitudes of the delivered forces have been estimated in vitro: similar Ni-Ti wires deliver forces in the range from 0.7 N to 1 N during the plateau region (deactivation) at a deflection of 1.5 mm [12].

In each volunteer, measurements of blood flow were taken in five sessions: (i) prior to the wire activation (recordings considered as basal flow, here denoted as session *before*) and after the placement of the wire, four other measurements were taken: (ii) twenty minutes after setting the wire (20 min); (iii) 48 hours after setting the wire (48 h); and (iv) 72 hours after setting the wire (72 h); (v) one month after setting the wire (Day 30).

### 2.5. Recording Procedures

Three measurements of blood flow (*F*_1_, *F*_2_, and *F*_3_) were taken during each session, each one lasting one minute. In each of these measurements, the average value was taken, excluding artifacts caused by involuntary movements. The results from the three measurements of blood flow were averaged: *F* = (*F*_1_ + *F*_2_ + *F*_3_)/3. 

### 2.6. Indicative Parameter of Alterations of Blood Flow

The data obtained from LDF in AU were analyzed according to the formula

(1) $$\begin{array}{c} F\left(\text{diff}\right)=F_{B}-F_{A}, \end{array}$$

where, for each tooth, *F*_*B*_ is the initial (basal) flow in AU and *F*_*A*_ are the values from the following testing sessions in AU (20 min, 48 h, 72 h, and day 30). By definition, during the session before (basal flow), *F*(diff) = 0. 

### 2.7. Stability of Records

Aiming to evaluate the stability of measurements of BFS in this study, the blood flow of five maxillary left central incisors from five individuals (a subgroup from the above described group, with mean age of 17 ± 3.9 years) was measured to determine whether there were any significant variations in the blood flows when there were no forces delivered to the teeth. Measurements were taken in two sessions: one at a certain time and the second one month later, prior to the treatment (without forces). 

### 2.8. Statistical Analyses

Data normality was verified. Standard descriptive statistics and ANOVA for repeated measures were used to assess the effect of the forces on the differences of flows in the studied sessions during the alignment and leveling phase. Sphericity tests were performed. Multiple comparisons were performed using the Tukey method to verify which sessions differed. The stability of the records was analyzed using paired samples Student's *t*-test. Values of *P* < 0.05 indicate statistical significance.

## 3. Results

### 3.1. Alterations in Blood Flow in the Alignment and Leveling Phase

**Figure 4:**
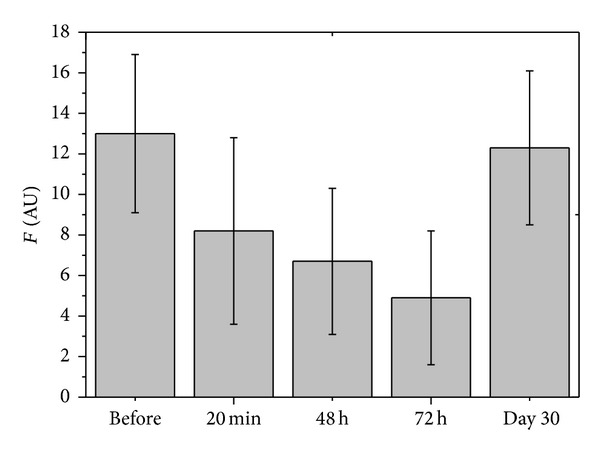
Mean values of BFS from 12 subjects in arbitrary units (AU) during the studied sessions. Vertical bars are standard deviations.

The mean values of the measured BFS are shown in Figure 4, and the corresponding mean values of the differences of flow according to (1) are shown in Table 1. As can be verified in Figure 4 and Table 1, after the forces were applied, the mean values were reduced in the sessions at 20 min, 48 h, and 72 h, followed by a recovery on day 30. The ANOVA for repeated measures resulted in differences in responses (*P* < 0.001). Multiple comparisons with the Tukey's test show that the responses in the sessions at 20 min, 48 h, and 72 h, although not statistically different among them, were all different from the initial session (session before) and from the session on day 30 (*P* < 0.05), as shown in Table 1. No difference was found comparing the basal flow (session before) and flow during the session on day 30.

### 3.2. Stability of Records

The data (stability of records) collected during two sessions 30 days apart, as explained above, resulted in means values and standard deviations equal to 15.7 ± 5 AU and 14.8 ± 4.2 AU, respectively. No statistically significant differences were found (paired Student's *t*-test, *P* = 0.24).

## 4. Discussion

### 4.1. Effects of the Forces on BFS

Reduced values of BFS were observed immediately after the forces were applied (20 minutes later), in accordance with other reports [5, 7–9]. Reduced values of BFS were also observed at 48 hours and 72 hours, in accordance with the results obtained by Sano et al. [8]. The flow on day 30 was not studied in the above mentioned studies, and fluxes at 48 hours and 72 hours were studied only by Sano et al. [8]. There are, at least, two possibilities justifying reduced flow, as follows.

A possibility to explain a decrease in the BFS immediately after the application of forces (20 min), as observed in this study, could be the constriction of vessels that enter and leave the apical foramen through the action of the dental dislocation. When afferent vessels are strangled, arteriolar resistance increases and the flow decreases. When efferent vessels are constricted, the venular resistance is increased. So, in these two scenarios the afferent flow is decreased (ischemia), and/or the efferent flow is decreased (passive hyperemia). Such a mechanism is also a first possibility to explain the decrease in flow as long as forces are active (i.e., at 48 hours and 72 hours). 

In the first stage of the treatment, a common and necessary reaction is an inflammatory process in the periodontal support tissues. In addition, it has been suggested that the entire complex: periodontal ligament, alveolar bone, and pulp react as a whole, and a pulpal inflammation may be present [1]. In other words, a second possibility to explain a further decrease in the BFS is an inflammatory process in the pulp.

In an inflammatory process, vasodilatation and increased vascular permeability are initial reactions. In the pulpal chamber, both these reactions cause an increase in the interstitial pressure due to the increased volume of pulpal fluid. Due to the low compliance of the pulpal environment, it has been speculated that the increased interstitial pressure could result in a vascular compression thereby reducing blood flow [13]. The drainage of the interstitial fluid, via lymphatic and capillary vessels, could also occur [14]. In this case, the interstitial pressure would not increase to very high levels, resulting in a more favorable course for the reparation of the pulp. During orthodontic forces, however, one can speculate that drainage may be diminished because the pressure of lymphatic vessels and venules is low, and as a result these vessels are compressed. In addition, mechanical compression of these vessels due to the tooth displacement also reduces the drainage. Thus, one could expect reduced pulpal flow during the action of orthodontic forces. 

The wire studied was a superelastic nickel-titanium alloy, designed to produce almost constant forces over a wide range of displacements, delivering light forces to the teeth [12, 15]. 

Experimental simulations of (human) clinical conditions have demonstrated that 0.014-inch superelastic archwires produce almost constant forces in the plateau region. However, the resulting pick forces prior to the plateau, just after the wire is loaded, can be up to (approximately) 9 N [12]. Indeed, it has been speculated that superelastic forces lead to an efficient movement but with same damage in the periodontium [16]. Thus, considering the initial heavy force that could be applied, one or both of the above conjectured mechanisms (compression of vessels and inflammation) could have occurred during the treatment. 

The values of BFS on day 30 were close to the basal values (session before), suggesting that the forces were already decaying (or decayed) and any inflammatory process that occurred during the initial phase was reversing (or reversed). However, further research, considering more samples and a longer interval of study, is necessary to clarify this issue.

It is known that BFS contain periodontal signals. Therefore, in this sequence, the periodontal vascular responses to orthodontic forces are also discussed. 

Decreased blood volume was observed in human periodontal ligaments (PDL) using a real-time method (optical plethysmography), immediately after compression by orthodontic forces ranging from (approximately) 30 g to 480 g. In the tensioned zone of the PDL, the response observed with forces of up to 90–180 g was increased blood volume. Above this range, the observed response was an initial increase in blood volume followed (immediately or after few seconds) by a decreased blood volume [17]. In a later study in rat's incisors, using vital microscopy, ischemic areas in both compressed and tensioned zones in the PDL were observed, beginning in capillaries and venules in response to 0.03 N and ending with arterioles (0.1 N). After continuous force of 0.1 N during 60 min, irreversible thrombosis was observed [18]. As mentioned above, superelastic wires can deliver heavy forces prior to the plateau region and from light to moderate forces in the plateau region. Thus, decreased blood flow in the PDL at 20 min, 48 h, and 72 h is a justified possibility. 

### 4.2. Measurement Method: Probe Stabilization, Tooth Isolation, and Contamination

For noninvasive measurements of pulpal blood flow via laser Doppler flowmetry, an optical fiber delivers the laser radiation to the tooth enamel. A fraction of the transmitted radiation, by the enamel and dentin, reaches the pulp, and via an inverse pathway the backscattered radiation is collected by another optical fiber for signal detection and processing. It is recognized that a fraction of the scattered radiation reaches also the periodontal tissues and a fraction of the measured flow via LDF originates from these regions [8, 19–21].

Aiming to reduce the contaminating fraction from the gingiva, the use of a rubber dam, acting as an optical isolator (or optical barrier), placed onto the gingival surface to shield the gingival region, has been suggested [8, 19–22]. It is recognized that the rubber dam not only acts as an optical isolator but also compresses the gingival region, reducing the regional blood flow [8, 19, 20]. As will be seen below, alterations in pressure could result in alterations in the fraction of contamination. In this study, a rubber dam was not used because it is difficult to control the resulting compression when a fixed orthodontic appliance is present. This procedure could increase the variability in the measurements because the resulting compression could vary in each session, consequently varying the fraction of contamination from the gingival regions. There are other methods designed to reduce the contamination, including the isolation of multiple teeth using a silicone splint [11], as the one used in this study. 

In order to further minimize the contaminations, the cutoff frequency of the LDF was set at 3.1 KHz (instead of 15 kHz which is most commonly used, e.g., [8, 9, 21, 22]) to reduce the interferences from nonpulpal regions because the larger vessels at the gingival region produce higher Doppler shifts. Thus, a lower cutoff frequency has been suggested to reduce contaminations, [23].

Most commonly used probe holders were made from transparent or semitransparent materials [21, 22] or an acrylic disc without any isolation over the crown [8]. Experiments in pigs demonstrate that a reflexive encapsulation of the tooth increases the pulpal signal without increasing nonpulpal signals [24]. In accordance with this result, Hartman et al. [22] obtained significantly higher values of BFS from a silicone holder than from a semitransparent polyurethane holder. The silicone splint used in this study is an efficient diffuse reflector, reflecting back the radiation scattered by the dentin that is lost when transparent holders are used, probably increasing the pulpal signal without increasing nonpulpal signals. 

In order to further minimize the gingival contamination, processing the difference of flow is proposed (see (1)). The rationales are as follows. 

During the measurements, the gingival region would be compressed by the walls of the silicone splint. In addition, the gingiva was compressed or stretched, due to the orthodontic forces.

It has been reported that, under pressure, the gingival blood flow is reduced. After the force is removed, the blood flow is increased (hyperemia), returning to the baseline level in few minutes [25, 26]. As mentioned above, it is not possible to use a punched rubber dam when using fixed orthodontic appliances, and it is difficult to adjust the pressure of a strip of rubber as proposed by Sano et al. [8]. In order to avoid any fluctuation of the compression and the consequent fluctuation in the measured flow, in this study, a silicone splint was used as optical barrier and probe holder. Care was taken to fit the silicone to the gingival region without causing excessive compression. 

Admitting the measured flow, (*F*), to be composed by fractions of flow from pulpal (*P*) and nonpulpal (*NP*) regions, then the measured flow can be expressed as *F* = *P* + *NP* [27]. Similarly, consider that the nonpulpal fraction is formed by the gingival and other periodontal fractions, *NP* = *G* + *Pe*, where *G* and *Pe* are the gingival and (other) periodontal fractions. Thus, the flow can be expressed as *F* = *P* + *G* + *Pe*. Then, the difference of flow from two consecutive measurements (ideally) cancels the gingival fraction because this fraction remained (ideally) invariable, while the pulpal and periodontal fractions vary largely; that is, *F*_2_ − *F*_1_ = (*P*_2_ − *P*_1_) + (*G*_2_ − *G*_1_) + (*Pe*_2_ − *Pe*_1_) = (*P*_2_ − *P*_1_) + (*Pe*_2_ − *Pe*_1_) where numerical subscripts means two measurements at different times. Yamaguchi et al. [28] demonstrated that orthodontic forces in the order of 0.7 N reduced gingival flow immediately after forces were applied followed by a recovery at ten minutes. Thus, one can expect transitory alterations in gingival blood flow immediately after the forces are applied, followed by a recovery. In our experiment, transitory alterations were avoided because measurements were performed at least 20 minutes after the forces were applied. 

### 4.3. Stability of the Records

The method here described allowed repetitive measurements up to 30 days. The following factors, the method used for probe positioning (using the bracket as a reference), performing three measurements of flow during each session, the reduced cutoff frequency in the LDF (3.1 kHz), the silicone splint (opaque diffuser and isolator) used, and the method for signal processing (difference of flow) all contribute to minimize variability in the measurements.

### 4.4. Implication of the Results and Limitations of This Study

The reduced BFS observed at 20 minutes, 48 hours, and 72 hours after forces were applied may be associated with inflammatory processes, justifying iatrogenic alterations as a consequence of orthodontic forces [1]. As there are few investigations performed in this regard, further studies are necessary to confirm the reactions of pulpal and adjacent tissues that might occur when mechanical forces produced by orthodontic appliances are used. 

## 5. Conclusion

Under clinical conditions, the orthodontic forces produced by the tested superelastic Ni-Ti wire significantly reduced the blood-flow signals measured by a laser Doppler flowmeter, at 20 minutes, 48 hours, and 72 hours after forces were applied. On day 30 after forces activation, the measured blood flow signals were close to the basal values. 

## Figures and Tables

**Table 1:** Mean values (*n* = 12) and standard deviations (SD) of differences of flow during the evaluated sessions.

	Before	20 min	48 h	72 h	day 30
*F*(diff) (AU)	0	−4.9*	−6.3*	−8.1*	−0.7
SD (AU)	0	3.7	4.8	4.1	4.4

*Means statistical differences when compared to sessions “before” and “day 30” (Anova, Tukey's test, *P* < 0.05). Values are expressed in arbitrary units (AU).

## References

[1] Vandevska-Radunovic V (1999). Neural modulation of inflammatory reactions in dental tissues incident to orthodontic tooth movement. A review of the literature. *European Journal of Orthodontics*.

[2] Villa PA, Oberti G, Moncada CA (2005). Pulp-dentine complex changes and root resorption during intrusive orthodontic tooth movement in patients prescribed nabumetone. *Journal of Endodontics*.

[3] Yamaguchi M, Kasai K (2007). The effects of orthodontic mechanics on the dental pulp. *Seminars in Orthodontics*.

[4] Oppenheim A (1942). Human tissue response to orthodontic intervention of short and long duration. *American Journal of Orthodontics and Oral Surgery*.

[5] Brodin P, Linge L, Aars H (1996). Instant assessment of pulpal blood flow after orthodontic force application. *Journal of Orofacial Orthopedics*.

[6] Barwick PJ, Ramsay DS (1996). Effect of brief intrusive force on human pulpal blood flow. *American Journal of Orthodontics and Dentofacial Orthopedics*.

[7] Ikawa M, Fujiwara M, Horiuchi H, Shimauchi H (2001). The effect of short-term tooth intrusion on human pulpal blood flow measured by laser Doppler flowmetry. *Archives of Oral Biology*.

[8] Sano Y, Ikawa M, Sugawara J, Horiuchi H, Mitani H (2002). The effect of continuous intrusive force on human pulpal blood flow. *European Journal of Orthodontics*.

[9] Mcdonald F, Pitt Ford TR (1994). Blood flow changes in permanent maxillary canines during retraction. *European Journal of Orthodontics*.

[10] Vongsavan N, Matthews B (1993). Some aspects of the use of laser Doppler flow meters for recording tissue blood flow. *Experimental Physiology*.

[11] Setzer FC, Challagulla P, Kataoka SHH, Trope M (2012). Effect of tooth isolation on laser Doppler readings. *International Endodontic Journal*.

[12] Hemingway R, Williams RL, Hunt JA, Rudge SJ (2001). The influence of bracket type on the force delivery of Ni-Ti archwires. *European Journal of Orthodontics*.

[13] Heyeraas KJ, Kvinnsland I (1992). Tissue pressure and blood flow in pulpal inflammation. *Proceedings of the Finnish Dental Society*.

[14] Heyeraas KJ, Berggreen E (1999). Interstitial fluid pressure in normal and inflamed pulp. *Critical Reviews in Oral Biology and Medicine*.

[15] Sander C, Sander FM, Sander FG (2006). The behaviour of the periodontal ligament is influencing the use of new treatment tools. *Journal of Oral Rehabilitation*.

[16] Noda K, Nakamura Y, Kogure K, Nomura Y (2009). Morphological changes in the rat periodontal ligament and its vascularity after experimental tooth movement using superelastic forces. *European Journal of Orthodontics*.

[17] Packman H, Shoher I, Stein RS (1977). Vascular responses in the human periodontal ligament and alveolar bone detected by photoelectric plethysmography: the effect of force application to the tooth. *Journal of Periodontology*.

[18] Gaengler P, Merte K (1983). Effects of force application on periodontal blood circulation. A vital microscopic study in rats. *Journal of Periodontal Research*.

[19] Soo-Ampon S, Vongsavan N, Soo-Ampon M, Chuckpaiwong S, Matthews B (2003). The sources of laser Doppler blood-flow signals recorded from human teeth. *Archives of Oral Biology*.

[20] Jafarzadeh H (2009). Laser Doppler flowmetry in endodontics. *International Endodontic Journal*.

[21] Kijsamanmith K, Timpawat S, Vongsavan N, Matthews B (2011). Pulpal blood flow recorded from human premolar teeth with a laser Doppler flow meter using either red or infrared light. *Archives of Oral Biology*.

[22] Hartmann A, Azérad J, Boucher Y (1996). Environmental effects on laser Doppler pulpal blood-flow measurements in man. *Archives of Oral Biology*.

[23] Mesaros S, Trope M, Maixner W, Burkes EJ (1997). Comparison of two laser Doppler systems on the measurement of blood flow of premolar teeth under different pulpal conditions. *International Endodontic Journal*.

[24] Vongsavan N, Matthews B (1996). Experiments in pigs on the sources of laser Doppler blood-flow signals recorded from teeth. *Archives of Oral Biology*.

[25] Baab DA, Oberg PA, Holloway GA (1986). Gingival blood flow measured with a laser doppler flowmeter. *Journal of Periodontal Research*.

[26] Patiño-Marín N, Martínez F, Loyola-Rodríguez JP, Tenorio-Govea E, Brito-Orta MD, Rodríguez-Martínez M (2005). A novel procedure for evaluating gingival perfusion status using laser-Doppler flowmetry. *Journal of Clinical Periodontology*.

[27] Roebuck EM, Evans DJP, Stirrups D, Strang R (2000). The effect of wavelength, bandwidth, and probe design and position on assessing the vitality of anterior teeth with laser Doppler flowmetry. *International Journal of Paediatric Dentistry*.

[28] Yamaguchi K, Nanda RS, Kawata T (1991). Effect of orthodontic forces on blood flow in human gingiva. *Angle Orthodontist*.

